# Selegiline Ameliorates Depression-Like Behavior in Mice Lacking the *CD157/BST1* Gene, a Risk Factor for Parkinson’s Disease

**DOI:** 10.3389/fnbeh.2017.00075

**Published:** 2017-05-03

**Authors:** Satoka Kasai, Toru Yoshihara, Olga Lopatina, Katsuhiko Ishihara, Haruhiro Higashida

**Affiliations:** ^1^Research Institute, FP Pharmaceutical CorporationMatsubara, Japan; ^2^Department of Basic Research on Social Recognition and Memory, Research Center for Child Mental Development, Kanazawa UniversityKanazawa, Japan; ^3^Institute of Laboratory Animals, Graduate School of Medicine, Kyoto UniversityKyoto, Japan; ^4^Department of Immunology and Molecular Genetics, Kawasaki Medical SchoolKurashiki, Japan

**Keywords:** *CD157/BST-1*, Parkinson’s disease, non-motor symptoms, selegiline, mirtazapine, forced swimming test, open field test

## Abstract

Parkinson’s disease (PD), a neurodegenerative disorder, is accompanied by various non-motor symptoms including depression and anxiety, which may precede the onset of motor symptoms. Selegiline is an irreversible monoamine oxidase-B (MAO-B) inhibitor, and is widely used in the treatment of PD and major depression. However, there are few reports about the effects of selegiline on non-motor symptoms in PD. The aim of this study was to explore the antidepressant and anxiolytic effects of selegiline, using *CD157/BST1* knockout (*CD157* KO) mouse, a PD-related genetic model displaying depression and anxiety, compared with other antiparkinsonian drugs and an antidepressant, and was to investigate the effects of selegiline on biochemical parameters in emotion-related brain regions. A single administration of selegiline (1–10 mg/kg) dose-dependently reduced immobility time in the forced swimming test (FST) in *CD157* KO mice, but not C57BL/6N wild-type (WT) mice. At 10 mg/kg, but not 3 mg/kg, selegiline significantly increased climbing time in *CD157* KO mice. A single administration of the antiparkinsonian drugs pramipexole (a dopamine (DA) D2/D3 receptor agonist) or rasagiline (another MAO-B inhibitor), and repeated injections of a noradrenergic and specific serotonergic antidepressant (NaSSA), mirtazapine, also decreased immobility time, but did not increase climbing time, in *CD157* KO mice. The antidepressant-like effects of 10 mg/kg selegiline were comparable to those of 10 mg/kg rasagiline, and tended to be stronger than those of 1 mg/kg rasagiline. After the FST, *CD157* KO mice showed decreases in striatal and hippocampal serotonin (5-HT) content, cortical norepinephrine (NE) content, and plasma corticosterone concentration. A single administration of selegiline at 10 mg/kg returned striatal 5-HT, cortical NE, and plasma corticosterone levels to those observed in WT mice. In the open field test (OFT), repeated administration of mirtazapine had anxiolytic effects, and selegiline nonsignificantly ameliorated anxiety-like behaviors in *CD157* KO mice. In the social interaction and preference tests, repeated mirtazapine ameliorated the high anxiety and low sociability of *CD157* KO mice, whereas selegiline did not. These results indicate that selegiline has antidepressant and mild anxiolytic effects in *CD157* KO mice, and suggest that it is an effective antiparkinsonian drug for depressive and anxiety symptoms in PD patients with a *CD157* single nucleotide polymorphism (SNP).

## Introduction

Parkinson’s disease (PD) is a progressive neurodegenerative disorder characterized by motor symptoms such as bradykinesia, rigidity, tremor at rest and postural instability, which arise mainly from dysfunction of the nigrostriatal dopaminergic pathway (Jankovic, 2008; Braak and Del Tredici, 2009). PD is also accompanied by non-motor symptoms including autonomic dysfunction, cognitive and psychiatric abnormalities (e.g., dementia, depression, apathy, anxiety and hallucination), sleep disorders and sensory abnormalities (Jankovic, 2008). Non-motor symptoms in PD correlate with advancing age and disease severity, although some non-motor symptoms including olfactory problems, constipation and depression, can occur early in the disease (Chaudhuri et al., 2006).

Depression and anxiety appear in approximately 40% (4%–70%) and 50% of all PD patients, respectively (Cummings, 1992; Leentjens et al., 2008). Non-motor symptoms greatly contribute to a reduced quality of life for patients with PD (Yamamoto, 2001; Edwards et al., 2002). The classic treatment L-3,4-dihydroxyphenylalanine (levodopa) has little effect on most non-motor symptoms (Chaudhuri et al., 2006), and chronic administration is associated with a risk of depression and anxiety (Damãsio et al., 1971; Marsh and Markham, 1973; Choi et al., 2000; Nègre-Pagès et al., 2010; Eskow Jeunarajs et al., 2011). There are some differences in neural circuitry dysfunction between major depression and PD-associated depression. For instance, suicidal tendencies and expression of guilt and self-blame are rarely observed in depressed PD patients, in contrast to patients with major depressive disorder (Brooks and Doder, 2001; Lemke, 2008; Starkstein et al., 2008). Postmortem studies of brains from PD patients have shown stage-dependent deposition of aggregated α-synuclein and neuronal loss in multiple brain areas such as serotonergic neurons in the raphe nuclei, noradrenergic neurons in the locus coeruleus, dopaminergic neurons in the substantia nigra and ventral tegmental area, and cortical neurons in regions interconnected with limbic structures (Braak et al., 2004). Conversely, brains from patients with major depression have reductions in gray-matter volume and glial density in the prefrontal cortex and hippocampus (Krishnan and Nestler, 2008), and dysfunction in the prefrontal–subcortical circuits including the amygdala, ventral striatum, hippocampus and dorsal raphe nucleus (Heller, 2016). One of the key pathophysiological mechanisms in major depression is impaired negative feedback control of the hypothalamic–pituitary–adrenal (HPA) axis, resulting in progressively unrestrained glucocorticoid release (Holsboer, 2000). Sustained elevation of glucocorticoid concentration under conditions of prolonged and severe stress, which may damage hippocampal neurons, is not observed in depressed PD patients (McEwen, 2000; Sapolsky, 2000). Although adequate treatment is needed for depression and anxiety in PD, their pathophysiology remains poorly understood.

The classical monoamine hypothesis of depression suggests that the disorder arises from a deterioration of noradrenergic/serotonergic function. In depressed PD patients, there is evidence that levels of norepinephrine (NE; Braak et al., 2004) and serotonin (5-HT; Kish et al., 2008) in the brain and/or cerebrospinal fluid are lower than in healthy people. Serotonergic and noradrenergic neuron dysfunction occurs even in the preclinical period of PD. Therefore, noradrenergic and serotonergic systems may play a significant role in the manifestation of depression and anxiety in PD. Some clinical studies suggested that tricyclic antidepressants such as desipramine and reboxetine (NE reuptake inhibitors), nortriptyline (an NE and 5-HT reuptake inhibitor) and citalopram (a selective 5-HT reuptake inhibitor) improved depressive symptoms in PD patients (Devos et al., 2008; Lemke, 2008; Menza et al., 2009). However, there was insufficient evidence to support the efficacy of antidepressants for the treatment of depression in PD (Liu et al., 2013a). Psychiatric symptoms in PD are still considered difficult to treat, possibly owing to concerns of exacerbation of parkinsonism by antidepressants (Leo, 1996). Therefore, a monotherapeutic agent to treat both motor and non-motor symptoms of PD would be a valuable therapeutic strategy in early PD, eliminating the risk of adverse drug interactions.

Bone marrow stromal cell antigen-1 (BST-1) was first isolated as a cell surface molecule that supported the cell growth of pre-B cells (Kaisho et al., 1994; Ishihara and Hirano, 2000) and clustered in CD157 in Leucocyte Typing VI after genetic cloning (Itoh et al., 1994; Muraoka et al., 1996; Okuyama et al., 1996; Ishihara et al., 1997). CD157/BST-1 is a member of the NADase/ADP-ribosyl cyclase family, to which CD38 also belongs (Hirata et al., 1994; Itoh et al., 1994; Ferrero et al., 1999; Ishihara and Hirano, 2000; Guse, 2005; Malavasi et al., 2006, 2008; Higashida et al., 2012; Lee, 2012). CD157/BST-1 has a variety of roles in the humoral immune response, neutrophil transmigration and hematopoietic stem cell support (Ishihara and Hirano, 2000; Funaro et al., 2004; Podestà et al., 2005; Malavasi et al., 2006; Mouchiroud et al., 2013). CD157/BST-1 is also involved in the pathogenesis of several diseases such as survival of B lymphocytes in rheumatoid arthritis, progression of leukemia, and metastasis of human ovarian carcinoma cells (Kaisho et al., 1994; Shimaoka et al., 1998; Ishihara and Hirano, 2000; Ortolan et al., 2010; Quarona et al., 2013; Lo Buono et al., 2014). Recently, genome-wide association studies and meta-analyses for PD identified intronic single-nucleotide polymorphisms (SNPs) in the *CD157/BST1* gene on the human chromosome 4p15 as a new susceptibility locus in Asian and European populations (Satake et al., 2009; Tan et al., 2010; Liu et al., 2011, 2013b; International Parkinson Disease Genomics Consortium et al., 2011; Saad et al., 2011; Simón-Sánchez et al., 2011; UK Parkinson’s Disease Consortium et al., 2011; Zimprich, 2011; Lill et al., 2012; Sharma et al., 2012). In our previous study, *CD157/BST1* knockout (*CD157* KO) mice showed depression-like behaviors in the forced swimming test (FST) and the tail suspension test, anxiety-like behaviors in the open field test (OFT), the light dark transition test and the elevated plus maze test, and impaired social behaviors in the social preference test, which in part resemble psychiatric symptoms observed in patients with PD (Jankovic, 2008; Kummer et al., 2008). In contrast, *CD157* KO mice did not show any degeneration of nigrostriatal dopaminergic neurons, any apparent motor dysfunction, or any alteration in dopaminergic neuron susceptibility to 1-methyl-4-phenyl-1,2,3,6-tetrahydropyridine (MPTP; Lopatina et al., 2014). Genetic and environmental factors may be needed to consider the real pathogenic role of *CD157* SNPs or deletion, as suggested by a previous study (Chen et al., 2014). And additionally, we cannot exclude the possibility that psychiatric phenotypes in *CD157* KO mice are related to autism spectrum disorder (ASD), because it was reported that *CD157/BST1* SNPs showed significant association with ASD (Yokoyama et al., 2015). Although further studies remain to be carried out in order to elucidate how *CD157* mutation or deletion contributes to pathogenic process of PD or psychiatric symptoms, our previous study suggests that young adult *CD157* KO mice are possibly a genetic rodent model for psychiatry symptoms associated with PD.

Selegiline, a selective and irreversible monoamine oxidase (MAO)-B inhibitor, is widely used for the treatment of PD (Birkmayer et al., 1977), as well as for major and atypical depression at higher doses to inhibit both MAO-A and -B activity (Varga and Tringer, 1967; Mann and Gershon, 1980; Birkmayer et al., 1984). MAO-B inhibitors block the metabolism of dopamine (DA), and increase DA concentration in the synaptic cleft (Youdim, 2013). DA induces motivation, reward and hedonic states, and plays an important role in neuropsychiatric disorders such as depression. Selegiline was reported to have antidepressant effects mediated by the activation of D1 and D2 receptors in normal and depressed mice (Shimazu et al., 2005; Amiri et al., 2016). Moreover, several studies have shown that selegiline enhances the expression of brain-derived neurotrophic factor in cultured murine astrocytes and in the anterior cingulate cortex of mice (Mizuta et al., 2000; Gyárfás et al., 2010), and prevents dopaminergic neurons from degeneration (Zhu et al., 2008; Youdim, 2013; Kong et al., 2015). In* de novo* PD patients, a double-blind, randomized, placebo-controlled clinical study demonstrated that selegiline monotherapy improved depression scores in the Hamilton Depression Rating Scale and the mental subscale of the Unified PD Rating Scale, and also improved motor scores (Allain et al., 1993). However, to our knowledge, there are no reports of the effects of selegiline in animal models of psychiatric symptoms of PD. In addition, it is not clear whether the antidepressant effect of selegiline reported in the above-mentioned clinical study is independent of its effect on motor symptoms.

Here, we investigated the effect of selegiline on depression- and anxiety-like behaviors in *CD157* KO mice, a PD-related genetic model, and on biochemical parameters in their emotion-related brain regions. Furthermore, to clarify whether antiparkinsonian drugs acting on dopaminergic system have commonly the antidepressant action, we compared effects of selegiline on depression-like behavior in *CD157* KO mice with those of the antiparkinsonian drugs rasagiline (another MAO-B inhibitor) and pramipexole (a D2/D3 receptor agonist). We demonstrated that selegiline exerted significant antidepressant effects in a dose-dependent manner and showed a tendency to improve anxiety-like behavior in *CD157* KO mice. The antidepressant effects of selegiline could be related to an enhancement in dopaminergic signaling, and normalization of dysfunction in the monoaminergic system and HPA axis.

## Materials and Methods

### Animals

C57BL/6N wild-type (WT) mice were obtained from Japan SLC Inc. (Shizuoka, Japan). *CD157* KO mice were as previously described (Itoh et al., 1998). WT and *CD157* KO colonies were maintained by crossbreeding WT and homozygous mutant mice, respectively. For experiments, *CD157* KO mice were obtained by breeding between homozygous mutant mice (Lopatina et al., 2014). WT and *CD157* KO mice were housed at the Institute for Experimental Animals, Advanced Science Research Center, Kanazawa University, under standard conditions (22°C; 12-h light/dark cycle, lights on at 8:45 a.m.) in standard mouse cages (300 × 160 × 110 mm) with sawdust bedding, and food and water *ad libitum*. Mice were single-housed for 5–7 days before the behavioral tests in order to control for environmental and social factor on behaviors, and the behavioral tests were conducted when the mice were 8 weeks old. This study was carried out in accordance with the Fundamental Guidelines for Proper Conduct of Animal Experiment and Related Activities in Academic Research Institutions under the jurisdiction of the Ministry of Education, Culture, Sports, Science and Technology of Japan. The protocol was approved by the Committee on Animal Experimentation of Kanazawa University (AP-143261).

### Drugs

Selegiline hydrochloride (FP Pharmaceutical Co., Osaka, Japan) was dissolved in saline and administered by single or repeated (daily for 3 days) subcutaneous (s.c.) injections at a dose of 1–10 mg/kg, which was the effective dose and treatment in PD and/or depression rodent models (Fredriksson et al., 1999; Shimazu et al., 2005). Rasagiline mesylate (Sigma-Aldrich, St. Louis, MO, USA) was dissolved in saline and administered in a single s.c. injection at a dose of 1 or 10 mg/kg in order to compare the antidepressant effects between these two MAO-B inhibitors (Finberg and Youdim, 2002; Youdim and Tipton, 2002). Pramipexole dihydrochloride (Sigma-Aldrich) was dissolved in saline and administered in a single s.c. injection at a dose of 1 mg/kg which was reported to be the effective dose and treatment in PD and/or depression rodent models (Maj et al., 1997; Siuciak and Fujiwara, 2004; Kitagawa et al., 2009; Bonito-Oliva et al., 2014). A noradrenergic and specific serotonergic antidepressant (NaSSA), mirtazapine (Sigma-Aldrich) was suspended in 0.2% Tween 80 solution and injected intraperitoneally (i.p.) at a dose of 1 mg/kg daily for 7 days, based on our preparatory experiments of the previous study (Lopatina et al., 2014). All drugs or saline were administered to mice in a volume of 10 mL/kg. Mice were subjected to behavioral tests 1 h after the last injection of selegiline, rasagiline, pramipexole or saline, or 30 min after the last injection of mirtazapine.

### Forced Swimming Test (FST)

The FST was performed according to the method originally described (Porsolt et al., 1977). Mice were placed individually in a cylinder (height 49 cm, diameter 15 cm) filled with water (25 ± 1°C) to a depth of 20 cm, for 6 min. After the initial 2 min of vigorous activity, the total duration of immobility during the last 4 min of the test was recorded. The duration of immobility was defined as the time during which the mouse floated passively, made no attempt to escape and showed only slow movements to keep its head above the water. The immobile state was analyzed using a digital video system and ANY-maze video tracking software (Stoelting, Wood Dale, IL, USA). The duration of climbing behavior (an emotion-related behavior) was defined as the time during which the mouse made forceful thrashing movements with its forelimbs against the walls of the cylinder during the full 6 min-video recording, and was measured manually with a stop watch by an unrelated observer (Lopatina et al., 2014).

### Plasma Corticosterone Concentration

Blood samples were collected by cardiac puncture from isoflurane-anesthetized mice 1 h after the FST or without the FST exposure, into EDTA-containing tubes. Blood samples were centrifuged for 15 min at 4°C and 1000× g, and plasma samples were stored at −80°C until assay. Plasma corticosterone concentrations were measured using corticosterone ELISA kits (Enzo Lifesciences, Farmingdale, NY, USA), according to the manufacturer’s instructions.

### Open Field Test (OFT)

The OFT was performed as described previously (Lopatina et al., 2014). The open field chamber consisted of a square wooden box (550 × 600 × 400 mm), with the inner surfaces covered with polypropylene sheets. The open field was divided into a center zone (300 × 300 mm) and periphery. First, a mouse was placed in the arena for 10 min (session 1), then returned to its home cage. In session 2, a novel non-social object (a wire cage, 70 × 90 × 70 mm, bars 5 mm apart) was placed in the center zone. The mouse was placed into the arena with the non-social object for 10 min, before being returned to its home cage. In session 3, a naïve male 8-week-old C57BL/6N mouse was placed under the wire cage. The test mouse was again placed in the arena for 10 min. The percentage of the time spent in the center zone, number of entries into the center zone, total distance traveled, and immobility time were analyzed using a digital video system and ANY-maze video tracking software. At the end of session 3, the test chambers were sprayed with 1% sodium hypochlorite and 70% ethanol and cleaned with paper towels. The time interval between sessions was 2–3 min.

### Determination of Monoamines and their Metabolite Content in Brain

Animals subjected to the FST or OFT were sacrificed by cervical dislocation after blood collection via cardiac puncture under isoflurane anesthesia, and their brains were removed rapidly after decapitation. The cortex, striatum, amygdala and hippocampus were dissected and stored at −80°C until neurochemical quantification. Tissues were homogenized with a microhomogenizer in 0.2 M perchloric acid containing isoproterenol (cortex, amygdala and hippocampus, 10 pg/μL; striatum, 100 pg/μL) as an internal standard. The homogenates were kept on ice for 30 min and centrifuged for 20 min at 4°C and 15,000× g, and the supernatants were passed through a 0.45 μm filter. All samples were stored at −80°C until high performance liquid chromatography (HPLC) measurement.

The tissue content of DA and its metabolites 3,4-dihydroxyphenylacetic acid (DOPAC), 3-methoxytyramine (3-MT) and homovanillic acid (HVA), 5-HT and its metabolite 5-hydroxyindoleacetic acid (5-HIAA), and NE were measured in an HPLC**-**electrochemical detector system (ECD-70, Eicom Co., Kyoto, Japan). Each 10 μL sample was injected into a C18 reverse-phase column (Eicompak SC-5ODS: 3.0 mm × 150 mm, Eicom) conditioned at 25°C. The mobile phase [0.1 M acetic acid–citric acid buffer (pH 3.5), 15% methanol, 190 mg/L sodium 1-octanesulfonate and 5 mg/L EDTA] was delivered at a flow rate of 0.5 mL/min. The applied potential was set to +750 mV vs. Ag/AgCl. The tissue content of the monoamines and their metabolites was calculated using standard curves and expressed as μg/g wet tissue.

### Statistical Analysis

Statistical analyses were performed using SPSS 23.0 (IBM Corp., Armonk, NY, USA). The data are expressed as the mean ± SEM. The saline-treated WT mice and the saline-treated *CD157* KO mice were compared using Levene’s test, followed by *post hoc* two-tailed Student’s *t*-tests. Two-way analysis of variance (ANOVA) was performed to examine the interaction between the effects of drugs or FST, and genotypes. In each genotype, comparisons between the saline- and drug-treated groups were evaluated using one-way ANOVA followed by Dunnett’s test. The difference was considered statistically significant at a value of *P* < 0.05.

## Results

### Effects of Selegiline on Depression-Like Behavior in *CD157* KO Mice

**Figure 1:**
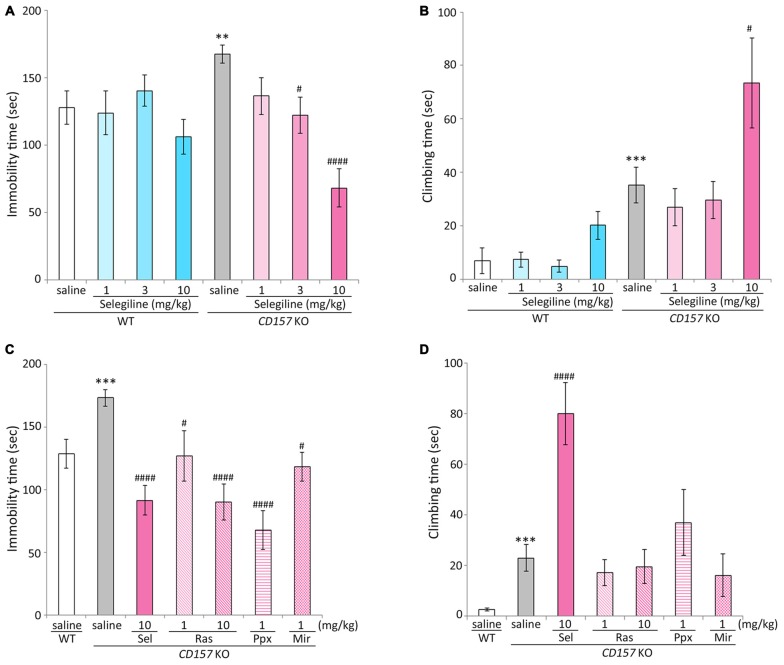
**Effects of monoamine oxidase-B (MAO-B) inhibitors, a dopamine (DA) agonist and a noradrenergic and specific serotonergic antidepressant (NaSSA) on a depression-like behavior in *CD157/BST1* knockout (*CD157* KO) mice subjected to the forced swimming test (FST). (A)** A single administration of selegiline (1−10 mg/kg, subcutaneous, s.c.) reduced immobility time of *CD157* KO mice in the FST. **P < 0.01, saline-treated *CD157* KO mice vs. saline-treated wild-type (WT) mice (Student’s *t*-test), ^#^*P* < 0.05, ^####^*P* < 0.001 vs. saline-treated *CD157* KO mice (Dunnett’s test), *F*_(3,64)_ = 12.539, *P* < 0.001. A two-way analysis of variance (ANOVA) showed a significant interaction between the effects of treatment and genotypes on the immobility time (*F*_(3,128)_ = 3.904, *P* < 0.05). **(B)** A single s.c. administration of selegiline at 10 mg/kg, but not 3 mg/kg, significantly increased climbing time of *CD157* KO mice. The data are expressed as the mean ± SEM (*n* = 21−22 for saline-treated WT and *CD157* KO mice, *n* = 15−17 for selegiline-treated WT and *CD157* KO mice). ***P < 0.005, saline-treated *CD157* KO mice vs. saline-treated WT mice (Student’s *t*-test), ^#^*P* < 0.05 vs. saline-treated *CD157* KO mice (Dunnett’s test), *F*_(3,67)_ = 4.382, *P* < 0.01. A two-way ANOVA showed no significant interaction between the effects of treatment and genotypes on the climbing time (*F*_(3,131)_ = 1.684, *P* = 0.174). **(C)** A single administration of selegiline (Sel; 10 mg/kg, s.c.), rasagiline (Ras; 1, 10 mg/kg, s.c.), or pramipexole (Ppx; 1 mg/kg, s.c.) and repeated administration of mirtazapine (Mir; 1 mg/kg, intraperitoneally, i.p.) reduced immobility time of *CD157* KO mice in the FST. ***P < 0.005, saline-treated *CD157* KO mice vs. saline-treated WT mice (Student’s *t*-test), ^#^*P* < 0.05, ^####^*P* < 0.001 vs. saline-treated *CD157* KO mice (Dunnett’s test), *F*_(5,86)_ = 10.276, *P* < 0.001. **(D)** Selegiline (10 mg/kg, s.c.) increased the climbing time of *CD157* KO mice, but rasagiline, pramipexole and mirtazapine did not. Data are expressed as mean ± SEM (*n* = 22−24 for saline-treated WT and *CD157* KO mice, *n* = 12−19 for drug-treated *CD157* KO mice). ***P < 0.005, saline-treated *CD157* KO mice vs. saline-treated WT mice (Student’s *t*-test), ^####^*P* < 0.001 vs. saline-treated *CD157* KO mice (Dunnett’s test), *F*_(5,87)_ = 8.471, *P* < 0.001.

Saline-treated *CD157* KO mice had significantly longer immobility times than saline-treated WT mice (*t* = −2.799, *P* < 0.01), indicating that *CD157* KO mice exhibited depression-like behavior, as previously described (Lopatina et al., 2014). The single administration of selegiline (1–10 mg/kg, s.c.) significantly reduced the immobility time of *CD157* KO mice in a dose-dependent manner (3 mg/kg, *P* < 0.05; 10 mg/kg, *P* < 0.001). However, a single administration of selegiline did not influence the immobility time of WT mice at any dose (Figure 1A). There were no differences in swimming speed between genotypes or treatment groups (Supplementary Figure).

Climbing time in saline-treated *CD157* KO mice was significantly longer than that in saline-treated WT mice (*t* = −3.465, *P* < 0.005). A single administration of selegiline at 10 mg/kg, but not 3 mg/kg, significantly increased climbing time in *CD157* KO mice (*P* < 0.05; Figure 1B).

### Effects of MAO-B Inhibitors, a DA Agonist and a NaSSA on a Depression-Like Behavior in *CD157* KO Mice

For the next set of experiment, we evaluated the effects of other monoaminergic compounds currently used in the treatment of PD and major depression on depression-like behavior in *CD157* KO mice. To evaluate the predictive validity and the involvement of DA signaling in depression-like behavior in *CD157* KO mice, we used 1 mg/kg pramipexole, which is an effective dose in animal models of PD and depression (Maj et al., 1997; Siuciak and Fujiwara, 2004; Kitagawa et al., 2009; Bonito-Oliva et al., 2014). Another MAO-B inhibitor, rasagiline, is reported to be 3–15 times more potent in MAO-B inhibition than selegiline in rats (Youdim et al., 2001), and has a levodopa equivalent dose (LED) one-tenth of that of selegiline in patients with PD (Tomlinson et al., 2010). In addition, there are some pharmacological differences in DA reuptake inhibition (Lamensdorf et al., 1999) and preferentially inducible neurotrophic factors (Naoi et al., 2013) between these MAO-B inhibitors. Therefore, 1 and 10 mg/kg rasagiline was selected for comparison with 10 mg/kg selegiline, which had shown effects on depression-like behavior and climbing (Figures 1A,B), as well as having antiparkinsonian effects in several rodent models (Fredriksson et al., 1999; Callizot et al., 2001; Leret et al., 2002; Rajendra Kopalli et al., 2012). Dosage of the NaSSA mirtazapine was 1 mg/kg i.p. daily for 7 days, at which it ameliorated depression- and anxiety-like behaviors in *CD157* KO mice, based on our preparatory experiments of the previous study (Lopatina et al., 2014).

Pramipexole (single injection of 1 mg/kg, s.c.) and mirtazapine (repeated injections of 1 mg/kg, i.p., daily for 7 days) reduced the elevated immobility time in *CD157* KO mice (pramipexole, *P* < 0.001; mirtazapine, *P* < 0.05). The antidepressant-like effect of 10 mg/kg selegiline was comparable to that of the same dose of rasagiline, and tended to be stronger than the effect of 1 mg/kg rasagiline (*t* = −1.602, *P* = 0.120; Figure 1C). There were no differences in swimming speed between treatment groups (data not shown). Selegiline (10 mg/kg, s.c.) increased climbing time in *CD157* KO mice (*P* < 0.001), whereas rasagiline, pramipexole and mirtazapine did not significantly influence climbing time (Figure 1D).

### Effects of Selegiline on Plasma Corticosterone Concentrations in *CD157* KO Mice Subjected to the FST

**Figure 2:**
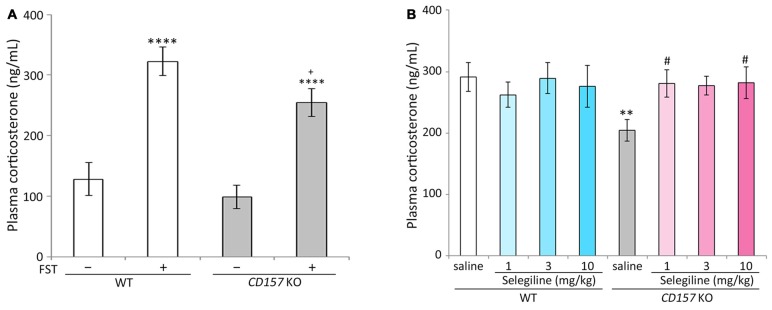
**Effects of selegiline on plasma corticosterone concentrations in *CD157* KO mice subjected to the FST. (A)** The FST induced a significant increase in plasma corticosterone concentrations in both WT and *CD157* KO mice, and plasma corticosterone concentrations in WT mice after the FST were higher than those of *CD157* KO mice. There were no significant differences in baseline plasma corticosterone concentrations between WT and *CD157* KO mice. Data are expressed as mean ± SEM (*n* = 11−16). ****P < 0.001 vs. non-treatment mice and ^+^*P* = 0.062 vs. WT after FST (Dunnett’s test). A two-way ANOVA showed main effects of FST (*F*_(1,49)_ = 50.861, *P* < 0.001) and genotype (*F*_(1,49)_ = 4.632, *P* < 0.05) without significant interaction between the effects of FST and genotypes on plasma corticosterone concentrations (*F*_(1,49)_ = 0.858, *P* = 0.359). **(B)** A single administration of selegiline (1−10 mg/kg, s.c.) elevated post-FST plasma corticosterone concentrations in *CD157* KO mice to the WT post-FST level. Data are expressed as mean ± SEM (*n* = 10−12). **P < 0.01, saline-treated *CD157* KO mice vs. saline-treated WT mice (Student’s *t*-test), ^#^*P* < 0.05 vs. saline-treated *CD157* KO mice (Dunnett’s test), *F*_(3,40)_ = 3.100, *P* < 0.05. Selegiline (3 mg/kg, s.c.) nonsignificantly elevated post-FST plasma corticosterone concentrations in *CD157* KO mice (*P* = 0.051). A two-way ANOVA showed no statistically significant interaction between the effects of selegiline and genotypes on plasma corticosterone concentration (*F*_(3,81)_ = 2.025, *P* = 0.117).

Stress adaptation failure is one of the primary neuropathological causes of depression (McEwen, 2000). Hyperactivity of the HPA axis, which results in elevated glucocorticoid levels, is consistently observed in depressed patients (Herman et al., 2003; de Kloet et al., 2005). In contrast, patients with PD and depression have significantly lower baseline levels of corticosterone than those with major depression alone, indicating that stress responses may be differentially regulated in these two patient populations (Pålhagen et al., 2010). We therefore measured plasma corticosterone concentrations in WT and *CD157* KO mice with or without FST exposure. There were no differences in baseline plasma corticosterone concentrations between WT and *CD157* KO mice. The FST induced significant increases in plasma corticosterone concentrations in both genotypes (WT mice, *P* < 0.001; *CD157* KO mice, *P* < 0.001). Interestingly, plasma corticosterone concentrations in *CD157* KO mice subjected to the FST were lower than those in WT mice (*P* = 0.062, Figure 2A; *t* = 2.874, *P* < 0.01, Figure 2B).

The single administration of selegiline (1 or 10 mg/kg, s.c.) 1 h before the FST significantly elevated post-FST plasma corticosterone concentrations in *CD157* KO mice, to levels comparable to those in WT mice (*P* < 0.05). In contrast, selegiline (1–10 mg/kg, s.c.) did not influence the FST-induced increase in plasma corticosterone in WT mice (Figure 2B).

### Effects of Selegiline and Mirtazapine on Anxiety-Like and Social Behaviors in *CD157* KO Mice

**Figure 3:**
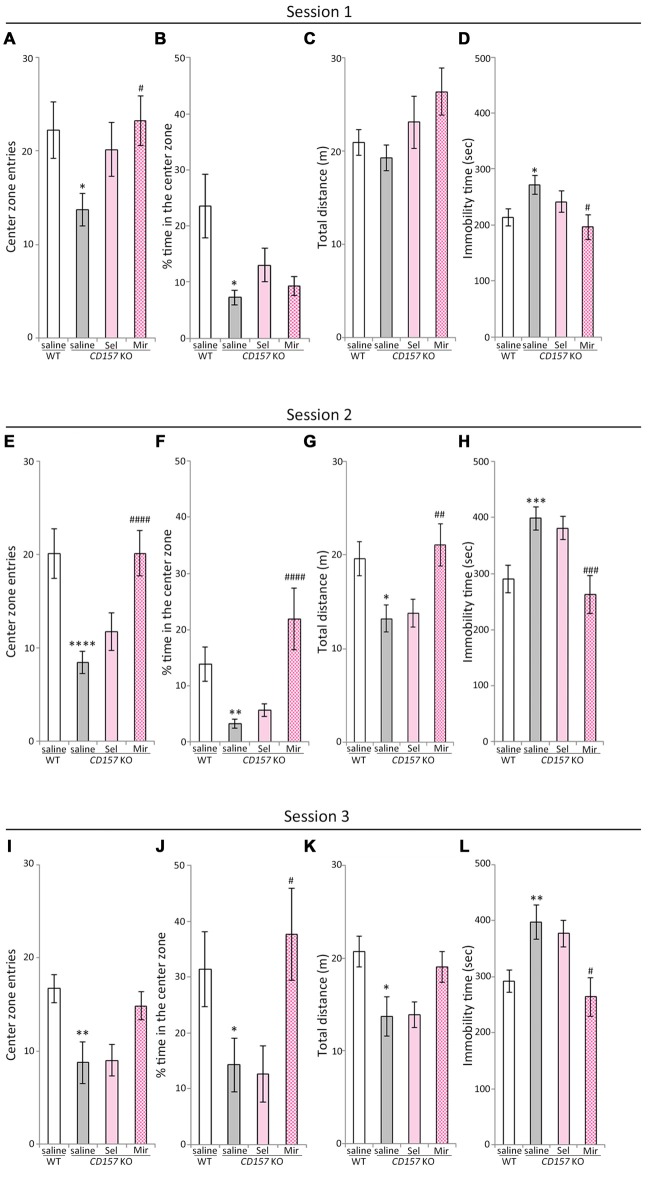
**Effects of selegiline and mirtazapine on anxiety-like and social behaviors in *CD157* KO mice. (A−D)** In session 1 (novel environment), *CD157* KO mice showed anxiety-like behavior compared with WT mice: significantly fewer entries into the center zone **(A)** and less time spent in the center zone **(B)**. Repeated administration of selegiline (1 mg/kg, s.c.) for 3 days showed a tendency to increase the number of entries into the center zone **(A)** in *CD157* KO mice. Repeated administration of mirtazapine (1 mg/kg, i.p.) for 7 days significantly increased the number of entries into the center zone **(A)**. There were no differences in measured values between genotypes and treatments in total distance traveled **(C)**. Immobility time in *CD157* KO mice was longer than that in WT mice. Mirtazapine reduced immobility time of *CD157* KO mice to that of WT mice **(D)**. **(E−H)** In session 2 (non-social target), *CD157* KO mice showed a significant decrease in the number of entries into the center zone **(E)**, percentage of time spent in the center zone **(F)** and total distance traveled **(G)** and a significant increase in immobility time **(H)** Mirtazapine, but not selegiline, significantly increased the number of entries into the center zone **(E)** and percentage of time spent in the center zone **(F)** in *CD157* KO mice. Mirtazapine significantly increased the total distance traveled **(G)**, and reduced prolonged immobility time **(H)** in saline-treated *CD157* KO mice. **(I−L)** In session 3 (social target), *CD157* KO mice showed significantly lower sociability than WT mice: significant decreases in the number of entries into the center zone **(I)**, percentage of time spent in the center zone **(J)** and total distance traveled **(K)**, and a significant increase in immobility time **(L)**. Mirtazapine significantly increased the percentage of time spent in the center zone **(J)** and decreased immobility time **(L)**, whereas selegiline did not alter the low-social behavior of *CD157* KO mice **(I−L)**. Data are expressed as mean ± SEM (*n* = 11−12 for saline-treated WT and *CD157* KO mice and selegiline-treated KO mice, *n* = 7−8 for mirtazapine-treated *CD157* KO mice). *P < 0.05, **P < 0.01, ***P < 0.005, ****P < 0.001, saline-treated *CD157* KO mice vs. saline-treated WT mice (Student’s *t*-test); ^#^*P* < 0.05, ^##^*P* < 0.01, ^###^*P* < 0.005, ^####^*P* < 0.001 vs. saline-treated *CD157* KO mice (Dunnett’s test). **(A)** *F*_(2,29)_ = 3.711, *P* < 0.05; **(B)** *F*_(2,29)_ = 1.874, *P* = 0.172; **(C)** *F*_(2,29)_ = 2.227, *P* = 0.126; **(D)** *F*_(2,29)_ = 3.537, *P* < 0.05; **(E)** *F*_(2,29)_ = 9.018, *P* < 0.005; **(F)** *F*_(2,29)_ = 13.980, *P* < 0.001; **(G)** *F*_(2,29)_ = 5.932, *P* < 0.01; **(H)** *F*_(2,29)_ = 8.249, *P* < 0.005; **(I)** *F*_(2,28)_ = 2.397, *P* = 0.109; **(J)** *F*_(2,28)_ = 4.810, *P* < 0.05; **(K)** *F*_(2,28)_ = 2.188, *P* = 0.131; **(L)** *F*_(2,28)_ = 4.804, *P* < 0.05.

The OFT, which measures approach to or avoidance of a central area is a commonly used and pharmacologically validated test for evaluating anxiety in a novel environment (Ramos and Mormède, 1998). In our previous studies, *CD157* KO mice displayed severe anxiety-like behaviors in the novel environment and in the presence of a novel non-social object, and a weak sociability against a social target in the OFT, and an abnormal sociability in a three-chamber paradigm (Lopatina et al., 2014; Mizuno et al., 2015; Higashida et al., 2017). We therefore evaluated the effects of repeated administration of selegiline and mirtazapine on anxiety-like behaviors, including sociability-related anxiety and social preference tasks in WT and *CD157* KO mice, using the OFT (Figure 3). In session 1, the number of entries into the center zone, and the percentage of time spent in the center zone, were lower in saline-treated *CD157* KO mice than in saline-treated WT mice (number of entries, *t* = 2.449, *P* < 0.05; percentage of time spent, *t* = 2.770, *P* < 0.05; Figures 3A,B), confirming the anxiety-like behavior in *CD157* KO mice described previously (Lopatina et al., 2014). There were no differences between genotypes or treatment groups in total distance traveled (Figure 3C). *CD157* KO mice that received repeated selegiline (1 mg/kg daily for 3 days) showed a tendency toward a higher number of entries into the center zone than those that received saline (*P* = 0.111; Figure 3A), suggesting that selegiline, administered repeatedly, has a tendency to reduce the elevated anxiety levels in *CD157* KO mice placed in a novel environment. The single administration of selegiline (1–10 mg/kg, s.c.), however, did not influence anxiety-like behaviors in *CD157* KO mice (data not shown), suggesting that repeated exposure to selegiline may be required for its anxiolytic effect. Repeated administration of mirtazapine (1 mg/kg daily for 7 days) resulted in a significant increase in the number of entries into the center zone, and a decrease in immobility time of *CD157* KO mice (number of entries, *P* < 0.05; immobility time, *P* < 0.05; Figures 3A,D), but failed to increase the percentage of time the *CD157* KO mice spent in the center zone (Figure 3B). These results indicated that repeated administration of mirtazapine improved anxiety-like behavior in *CD157* KO mice in the novel environment at the dose at which antidepressant-like effects were exerted.

Next, we performed the social interaction and preference tests in the same apparatus. In session 2 (non-social target) and session 3 (social target), saline-treated *CD157* KO mice showed significant decreases in the number of entries into the center zone and the percentage of time spent in it, as well as in the total distance traveled, in comparison with saline-treated WT mice (number of entries, *t* = 4.167, *P* < 0.001, Figure 3E;* t* = 2.978, *P* < 0.01, Figure 3I; percentage of time spent, *t* = 3.344, *P* < 0.01, Figure 3F;* t* = 2.125, *P* < 0.05, Figure 3J; total distance traveled, *t* = 2.771, *P* < 0.05, Figure 3G;* t* = 2.775, *P* < 0.05, Figure 3K). Immobility time in saline-treated *CD157* KO mice was significantly longer than in saline-treated WT mice (*t* = −3.458, *P* < 0.005, Figure 3H;* t* = −3.023, *P* < 0.01, Figure 3L). These results indicate that *CD157* KO mice exhibit higher levels of anxiety toward the novel non-social and social objects, as well as social avoidance. In session 2 (non-social target), repeated administration of mirtazapine significantly increased the number of the entries into the center zone (*P* < 0.001; Figure 3E), the percentage of time spent in the center zone (*P* < 0.001; Figure 3F) and the total distance traveled (*P* < 0.01; Figure 3G), and reduced immobility time in *CD157* KO mice (*P* < 0.005; Figure 3H). In session 3 (social target), repeated administration of mirtazapine significantly increased the percentage of time spent in the center zone (*P* < 0.05; Figure 3J), decreased immobility time (*P* < 0.05; Figure 3L) in *CD157* KO mice. These findings suggest that repeated administration of mirtazapine at 1 mg/kg for 7 days ameliorates the excess anxiety and low sociability of *CD157* KO mice. In contrast, repeated administration of selegiline at 1 mg/kg for 3 days had no effect on elevated levels of anxiety and social avoidance (Figures 3E-L).

### Changes in DA, 5-HT, NE and their Metabolites in Several Brain Regions of WT and *CD157* KO Mice after the FST, and Effects of a Single Administration of Selegiline

**Figure 4:**
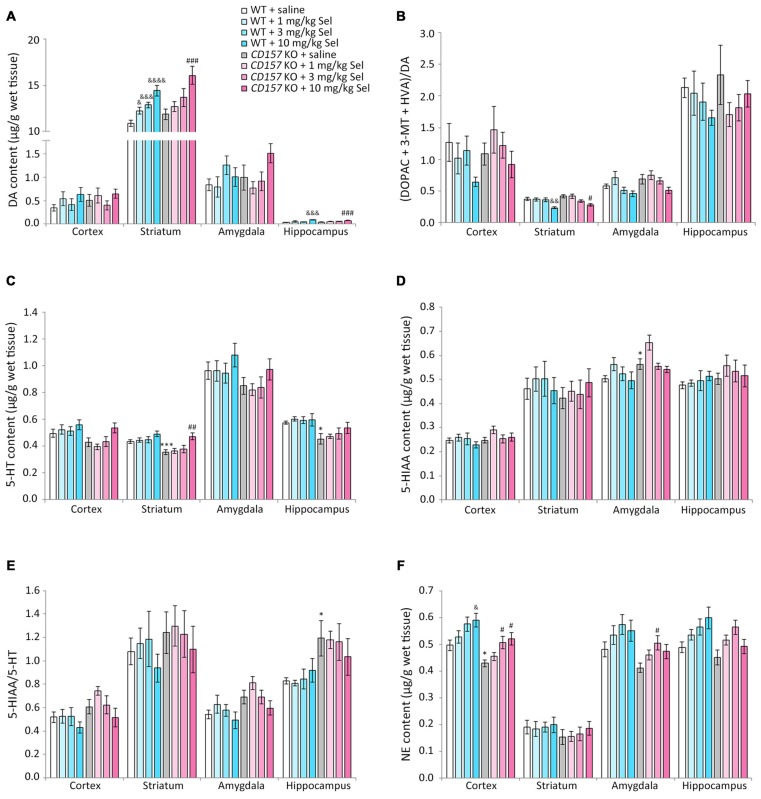
**Changes in DA, serotonin (5-HT), norepinephrine (NE) and their metabolites in several brain regions of WT and *CD157* KO mice after the FST, and effects of a single administration of selegiline.** Data are shown for DA **(A)**, ratio of DA metabolites to DA **(B)**, 5-HT **(C)**, 5-hydroxyindoleacetic acid (5-HIAA) **(D)**, 5-HIAA/5-HT ratio **(E)** and NE **(F)** in μg/g wet tissue (mean ± SEM; *n* = 6−9). *P < 0.05, ***P < 0.005, saline-treated *CD157* KO mice vs. saline-treated WT mice (Student’s *t*-test); ^&^*P* < 0.05, ^&&^*P* < 0.01, ^&&&^*P* < 0.005, ^&&&&^*P* < 0.001 vs. saline-treated WT mice (Dunnett’s test); ^#^*P* < 0.05, ^##^*P* < 0.01, ^###^*P* < 0.005 vs. saline-treated *CD157* KO mice (Dunnett’s test). **(A)** WT mice (striatum): *F*_(3,30)_ = 14.106, *P* < 0.001; *CD157* KO mice (striatum): *F*_(3,29)_ = 5.833, *P* < 0.005; WT mice (hippocampus): *F*_(3,30)_ = 5.315, *P* < 0.01; *CD157* KO mice (hippocampus): *F*_(3,29)_ = 6.113, *P* < 0.005; **(B)** WT mice (striatum): *F*_(3,30)_ = 5.708, *P* < 0.005; *CD157* KO mice (striatum): *F*_(3,29)_ = 3.531, *P* < 0.05; **(C)** *CD157* KO mice (striatum): *F*_(3,29)_ = 4.881, *P* < 0.01; **(F)** WT mice (cortex): *F*_(3,30)_ = 3.358, *P* < 0.05; *CD157* KO mice (cortex):*F*_(3,29)_ = 4.415, *P* < 0.05. A two-way ANOVA showed no significant interaction between the effects of treatment and genotypes on monoamine content or their metabolites in brain regions examined [cortex:* F*_(3,59)_ = 0.138, *P* = 0.937; striatum:* F*_(3,59)_ = 0.372, *P* = 0.774; amygdala:* F*_(3,56)_ = 1.127, *P* = 0.346; hippocampus: *F*_(3,59)_ = 0.927, *P* = 0.434, **(A)**; cortex:* F*_(3,59)_ = 0.662, *P* = 0.579; striatum: *F*_(3,59)_ = 0.832, *P* = 0.482; amygdala: *F*_(3,56)_ = 0.226, *P* = 0.878; hippocampus: *F*_(3,59)_ = 0.751, *P* = 0.526, **(B)**; cortex:* F*_(3,59)_ = 0.870, *P* = 0.462; striatum: *F*_(3,59)_ = 0.901, *P* = 0.446; amygdala: *F*_(3,56)_ = 0.028, *P* = 0.994; hippocampus: *F*_(3,59)_ = 0.466, *P* = 0.707, **(C)**; cortex:* F*_(3,59)_ = 0.683, *P* = 0.556; striatum: *F*_(3,59)_ = 0.308, *P* = 0.819; amygdala: *F*_(3,56)_ = 0.441, *P* = 0.724; hippocampus: *F*_(3,59)_ = 0.420, *P* = 0.739, **(D)**; cortex:* F*_(3,59)_ = 0.615, *P* = 0.608; striatum: *F*_(3,59)_ = 0.055, *P* = 0.983; amygdala: *F*_(3,56)_ = 0.157, *P* = 0.925; hippocampus: *F*_(3,59)_ = 0.671, *P* = 0.573, **(E)**; cortex: *F*_(3,59)_ = 0.004, *P* = 1.000; striatum: *F*_(3,59)_ = 0.069, *P* = 0.976; amygdala: *F*_(3,56)_ = 0.010, *P* = 0.999; hippocampus: *F*_(3,59)_ = 1.329, *P* = 0.274, **(F)**].

We evaluated the effects of selegiline on DA, 5-HT, and NE content, and their metabolites and turnover rates, in depression- and anxiety-related brain regions (cortex, striatum, amygdala and hippocampus) of WT and *CD157* KO mice after exposure to the FST (Figure 4). Saline-treated *CD157* KO mice had significantly lower levels of 5-HT in the striatum (*t* = 3.689, *P* < 0.005) and hippocampus (*t* = 3.052, *P* < 0.05; Figure 4C), and NE in the cortex (*t* = 2.795, *P* < 0.05; Figure 4F), than saline-treated WT mice, as well as higher levels of 5-HIAA in the amygdala (*t* = −2.399, *P* < 0.05; Figure 4D) without a significant increase in 5-HT turnover rate (Figure 4E). These data suggest that *CD157* KO mice have serotonergic and noradrenergic dysfunction in depression- and anxiety-related brain regions, but do not have dopaminergic dysfunction in the striatum, a region related to the motor symptoms of PD (Figures 4A,B).

Compared with saline, a single administration of selegiline (1–10 mg/kg, s.c.) increased DA content in the striatum in WT and *CD157* KO mice after the FST (WT mice: 1 mg/kg, *P* < 0.05; 3 mg/kg, *P* < 0.005; 10 mg/kg, *P* < 0.001; *CD157* KO mice: 10 mg/kg, *P* < 0.005) and in the hippocampus (WT mice: 10 mg/kg, *P* < 0.005; *CD157* KO mice: 10 mg/kg, *P* < 0.005; Figure 4A). In addition, striatal DA turnover (ratio of DA metabolites to DA) decreased in 10 mg/kg selegiline-treated WT (*P* < 0.01) and *CD157* KO mice (*P* < 0.05; Figure 4B). These data suggest that a single administration of selegiline produces increases in striatal DA content mediated by MAO-B inhibition. A single selegiline injection also dose-dependently increased cortical NE content (*P* < 0.05; Figure 4F). Furthermore, 10 mg/kg selegiline recovered the decreased striatal 5-HT content in *CD157* KO mice (*P* < 0.01; Figure 4C) without a significant decrease in 5-HIAA content and 5-HT turnover (Figures 4D,E). Thus, the antidepressant effects of selegiline may be mediated by enhancement of monoaminergic transmission.

### Changes in DA, 5-HT, NE and their Metabolites in Several Brain Regions of WT and *CD157* KO Mice after the OFT, and Effects of Repeated Administration of Selegiline and Mirtazapine

**Figure 5:**
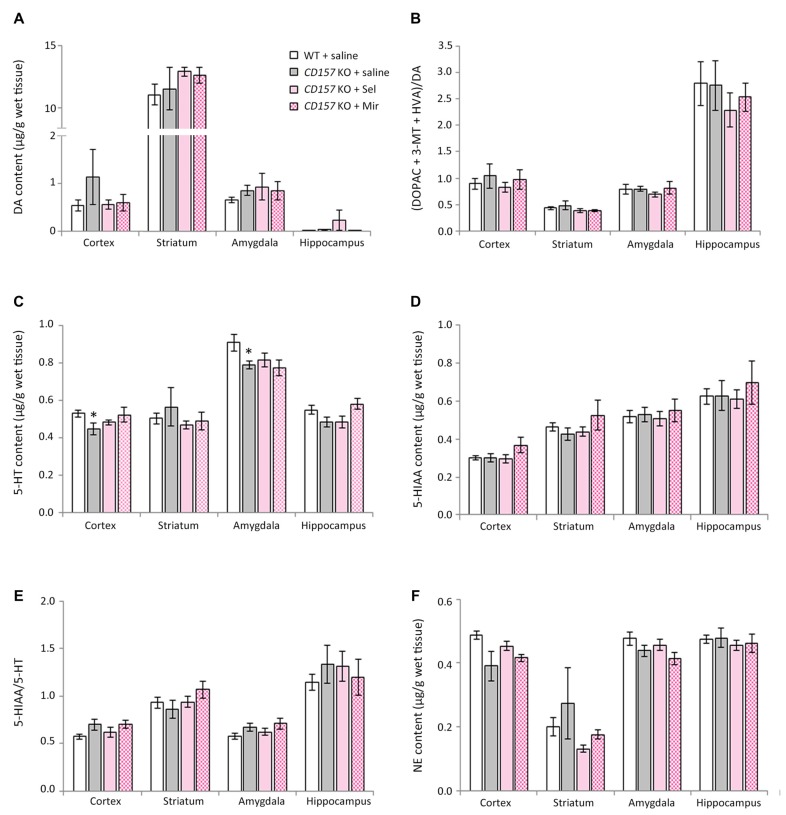
**Changes in DA, 5-HT, NE and their metabolites in several brain regions of WT and *CD157* KO mice after the open field test (OFT), and effects of repeated administration of selegiline and mirtazapine.** Data are shown for DA **(A)**, ratio of DA metabolites to DA **(B)**, 5-HT **(C)**, 5-HIAA **(D)**, 5-HIAA/5-HT ratio **(E)** and NE **(F)** in μg/g wet tissue as the mean ± SEM (*n* = 8 for saline-treated WT and *CD157* KO mice and selegiline-treated *CD157* KO mice, *n* = 4 for mirtazapine-treated *CD157* KO mice). *P < 0.05, saline-treated *CD157* KO mice vs. saline-treated WT mice (Student’s *t*-test).

We measured changes in DA, 5-HT and NE content, and their metabolites and turnover rates, in the cortex, striatum, amygdala and hippocampus of WT and *CD157* KO mice after exposure to the OFT (Figure 5). We also examined the effects of repeated administration of 1 mg/kg selegiline for 3 days and 1 mg/kg mirtazapine for 7 days. In saline-treated *CD157* KO mice, 5-HT content in the cortex and amygdala was significantly lower than in saline-treated WT mice (cortex: *t* = 2.248, *P* < 0.05; amygdala:* t* = 2.437, *P* < 0.05, Figure 5C). There were no differences in DA, NE and 5-HIAA content, and their turnover rates between genotypes or treatment groups (Figures 5A,B,D–F). Together, these data suggest that *CD157* KO mice have serotonergic dysfunction in different brain regions, regardless of the type of stress to which they are exposed.

Repeated administration of neither selegiline nor mirtazapine ameliorated the decreases observed in *CD157* KO mice in 5-HT content in the cortex and amygdala (Figure 5C).

## Discussion

Depression and anxiety are common non-motor symptoms in patients with PD, and have a detrimental effect on quality of life (Yamamoto, 2001; Edwards et al., 2002). Attributed to the monoaminergic dysfunction of PD, depression and anxiety in patients with PD are commonly treated with antidepressants for major depression. However, there is insufficient evidence for the efficacy and safety of this approach (Shabnam et al., 2003; Liu et al., 2013a). In the present study, after exposure to the FST, *CD157* KO mice had lower striatal and hippocampal 5-HT content and cortical NE content than WT mice. Administration of selegiline showed a recovery in forced swimming stress-induced decreases in striatal 5-HT and cortical NE concentrations in *CD157* KO mice to those in WT mice. Although no differences between genotypes were detected in DA content or turnover in the brain regions examined (cortex, striatum, amygdala and hippocampus) after the FST, the dopaminergic agents pramipexole, selegiline and rasagiline alleviated depression-like behavior in *CD157* KO mice. This suggests that depression-like behavior in *CD157* KO mice is associated with dopaminergic dysfunction in brain regions we did not examine, such as the mesolimbic pathway. Thus, these data suggest that forced swimming stress leads to serotonergic, and noradrenergic dysfunction in *CD157* KO mice, and that pharmacological modification of monoaminergic functions may exert antidepressant effects in *CD157* KO mice.

The enzyme MAO exists as two distinct subtypes, MAO-A and B, the former having a higher affinity for NE and 5-HT, and the latter for phenylethylamine. DA is a substrate of both enzymes. In *CD157* KO mice, the selective MAO-B inhibitor selegiline (3 and 10 mg/kg) significantly reduced immobility time in the FST, and at 10 mg/kg increased striatal and hippocampal DA content and decreased striatal DA turnover after the FST. Moreover, 10 mg/kg selegiline normalized FST-induced decreases in striatal 5-HT and cortical NE content after the FST in *CD157* KO mice. The antidepressant effects of selegiline in the FST may be mediated by the activation of D1 receptors (Shimazu et al., 2005; Amiri et al., 2016). Our data therefore suggest that the antidepressant effect of selegiline is attributable at least partially to the improvement of serotonergic, noradrenergic and dopaminergic dysfunction in *CD157* KO mice. From the point of view of effective doses of selegiline, its antidepressant efficacy would be greater in *CD157* KO mice than in WT mice or in the normal male ddY mice used by Shimazu et al. (2005). The difference in the effective doses of selegiline between the respective genotypes might arise from changes in monoaminergic dysfunction and/or expression of depression- and anxiety-associated proteins in *CD157* KO mice, suggesting that an effective dose of selegiline for the treatment of depression in PD may be lower than that in major depression.

Selegiline (3 and 10 mg/kg) significantly reduced immobility time in *CD157* KO mice, and at 10 mg/kg increased climbing time. Single administration of selegiline at 10 mg/kg did not influence the swimming speed of *CD157* KO mice in the FST (Supplementary Figure) or the total distance traveled in the OFT in *CD157* KO mice (data not shown). These data suggest that selegiline’s antidepressant effects do not necessarily result from increasing climbing behavior and are not elicited by stimulating motor activity, unlike stimulants such as methamphetamine, which reduces immobility time in the FST by causing hyperlocomotion (Kitada et al., 1981; Shimazu et al., 2005). Interestingly, treatment with rasagiline, pramipexole or mirtazapine did not increase climbing time in *CD157* KO mice, suggesting that the mechanisms underlying their antidepressant effects are different from those of selegiline. Several groups have demonstrated that NE reuptake inhibitors increase climbing behavior in the FST (Rénéric et al., 2002a,b; Cryan et al., 2005). In the present study, there was no significant correlation between cortical NE content in selegiline-treated *CD157* KO mice and their climbing time (data not shown), despite a dose-dependent increase in cortical NE content. Although a 50% inhibitory dose on striatal MAO-A activity is approximately 2.5 mg/kg for both selegiline and rasagiline in rats following i.p. administration (Youdim and Tipton, 2002), at 10 mg/kg, selegiline but not rasagiline significantly increased climbing time in *CD157* KO mice. Therefore, the increase in climbing time by selegiline might be related to an enhancement in noradrenergic transmission by other mechanisms such as monoamine reuptake inhibition (Lamensdorf et al., 1999) in brain regions we did not examine. It is widely accepted that climbing behavior in the FST is a specific activity aimed at escaping from the cylinder, and is one of the indexes for antidepressant-like behavior (Cryan et al., 2005; Perona et al., 2008), but we speculate that the increase in climbing time in *CD157* KO mice potentially reflects an altered emotional state (Lopatina et al., 2014). Therefore, the emotional state in which saline-treated *CD157* KO mice are motivated to try climbing may be qualitatively different from that in selegiline-treated *CD157* KO mice. Further investigation is required to clarify the meaning of climbing behavior in *CD157* KO mice.

Rasagiline is more potent as an MAO-B inhibitor than selegiline, and has a similar selectivity for MAO-B over MAO-A *in vivo* (Youdim et al., 2001). Its LED for motor symptoms in patients with PD is one-tenth of selegiline’s LED (Tomlinson et al., 2010). In *CD157* KO mice, the antidepressant-like effects of selegiline at 10 mg/kg had a tendency to be more potent than those of rasagiline at 1 mg/kg (*t* = −1.602, *P* = 0.120; Figure 1C), suggesting that selegiline is probably more effective for ameliorating depression in PD patients than rasagiline at doses that are therapeutically effective for the motor symptoms of PD. It also suggests that the MAO inhibition cannot entirely account for the antidepressant effects of selegiline. Our data are in line with clinical studies that selegiline improved Hamilton Depression Rating Scale scores in *de novo* PD patients (Allain et al., 1993), but rasagiline was not effective on Beck Depression Inventory scores (Barone et al., 2015).

There is insufficient evidence to support the efficacy of mirtazapine on depression and anxiety in PD patients, although its antidepressant effect in major depression is comparable to that of tricyclic antidepressants (Watanabe et al., 2011), which might be effective in PD-related depression (Devos et al., 2008; Lemke, 2008; Menza et al., 2009). In *CD157* KO mice, repeated administration of mirtazapine ameliorated depression-like behavior in the FST, and anxiety-like behavior and low sociability in the OFT. These results are consistent with the effects of mirtazapine in the tail suspension test and in the elevated plus maze test shown in our previous study (Lopatina et al., 2014). *CD157* KO mice showed decreases in 5-HT content in the cortex and amygdala, following the OFT, but mirtazapine failed to influence monoaminergic dysfunction in these brain regions in *CD157* KO mice. Therefore, mechanisms underlying anxiety and low sociability of *CD157* KO mice and anxiolytic effects of mirtazapine may be attributable principally to other monoaminergic systems, non-monoaminergic systems or neuroplasticity-associated protein expression (Ishima et al., 2014; Kadoguchi et al., 2014; Bittolo et al., 2016).

Like other inescapable stress paradigms, the FST elevates blood corticosterone concentrations in rodents (Steiner et al., 2008; Rogóz et al., 2012). Interestingly, plasma corticosterone concentrations after the FST were lower in *CD157* KO mice than in WT mice, although there were no significant differences in baseline levels between genotypes (Figures 2A,B). Surprisingly, following the FST in *CD157* KO mice, selegiline (1−10 mg/kg) normalized plasma corticosterone concentrations to those in WT mice (Figure 2B). Selegiline has a tendency to ameliorate dose-dependently the depression-like behavior and post-FST monoamine levels, but not post-FST corticosterone concentrations, in *CD157* KO mice, suggesting that such effect of selegiline on post-FST corticosterone concentrations in *CD157* KO mice is independent of enhancement of monoaminergic function in brain regions examined. Inputs from the amygdala elicit activation of the HPA axis (Sandi, 2004), and amygdala-lesioned rats show significantly lower plasma corticosterone and adrenocorticotropic hormone levels after certain types of stress than intact animals (Feldman and Conforti, 1981; Beaulieu et al., 1987). In our previous study, the amygdala in *CD157* KO mice seemed to be smaller than in WT mice (Lopatina et al., 2014); therefore, atrophy of the amygdala in *CD157* KO mice might be related to the reduction in plasma corticosterone concentrations following the FST. Moreover, amygdala dysfunction in *CD157* KO mice may also be related to their anxiety and depression-like behavior, because abnormalities in the amygdala might be involved in PD progression and may contribute to elicitation of non-motor symptoms such as anxiety and depression in PD (Huang et al., 2015; van Mierlo et al., 2015; Vriend et al., 2016). Thus, selegiline may ameliorate depressive symptoms by normalizing the hypoactivity of the HPA axis arising from the amygdala.

*CD157* KO mice had higher levels of anxiety in the novel environment (session 1) and toward a novel non-social object (session 2) than WT mice, confirming previous results (Lopatina et al., 2014; Mizuno et al., 2015). Moreover, when a novel object was placed in the apparatus (session 2), *CD157* KO mice showed a higher level of anxiety than in session 1 (percent of time in center zone in Figure 3B vs. Figure 3F, *t* = 2.794, *P* < 0.05). In the experiments with a social target (session 3), *CD157* KO mice seemed more nervous than WT mice, and showed weak sociability to the novel mouse, which is characteristic of their phenotype (Lopatina et al., 2014). Furthermore, their phenotype is possibly in line with clinical descriptions of social phobia occurring in 50% of PD patients (Kummer et al., 2008), and depressed PD patients having significantly fewer social ties than non-depressed PD patients (Starkstein et al., 1990). Repeated injection of selegiline at 1 mg/kg for 3 days had a tendency to improve the anxiety-like behavior of *CD157* KO mice in the novel environment (session 1), but did not alleviate the higher level of anxiety toward a novel non-social object (session 2) or the weak sociability to the novel social target (session 3). This suggests that selegiline improves mild anxiety but not severe anxiety or low sociability.

In the present study, there was a moderate negative correlation between 3-MT/DA ratio in the cortex and amygdala and immobility time in saline and selegiline-treated *CD157* KO mice in the FST (cortex, *r* = −0.496, *P* < 0.005; amygdala, *r* = −0.499, *P* < 0.01). At 10 mg/kg, selegiline increased the 3-MT/DA ratio in the cortex, amygdala and hippocampus, and reduced the DOPAC/DA ratio in the cortex, striatum and amygdala (data not shown), probably resulting from brain MAO-B inhibition. Intracerebroventricular administration of 3-MT increases behavioral activity (Nakazato and Akiyama, 2002; Sotnikova et al., 2010), and 3-MT and phenylethylamine have affinity for trace amine-associated receptor 1, which is involved in neuropsychiatric disorders including depression (Shi et al., 2016). Therefore, the antidepressant effect of selegiline might be mediated via MAO-B inhibition-induced enhancement of 3-MT and phenylethylamine content.

In conclusion, mice lacking a PD-related gene* CD157* show the depression- and anxiety-like behaviors and impairment of their brain monoamine content after exposure to stress. Selegiline exerted some anxiolytic effects in addition to antidepressant effects in *CD157* KO mice. These results highlight the potential of selegiline as an antiparkinsonian agent with the efficacy in *CD157* mutation-related depressive and anxiety symptoms.

## Author Contributions

HH, TY, OL and SK conceived and designed the research. SK performed experiments, analyzed data, and prepared the initial draft; HH and KI revised the manuscript. All authors reviewed the final manuscript and approved its publication.

## Funding

This work was supported by a grant-in-aid from “Integrated research on neuropsychiatric disorders” carried out under the Strategic Research Program for Brain Sciences. It was also supported by the Industry–Academia Collaborative R&D Program from the Ministry of Education, Culture, Sports, Science and Technology of Japan, and was a collaborative research project with FP pharmaceutical corporation.

## Conflict of Interest Statement

SK is an employee of FP Pharmaceutical Corporation. The other authors declare that the research was conducted in the absence of any commercial or financial relationships that could be construed as a potential conflict of interest.
