# Supplementary material for: Selegiline Ameliorates Depression-Like Behavior in Mice Lacking the CD157/BST1 Gene, a Risk Factor for Parkinson’s Disease
Source: Front Behav Neurosci. 2017 May 3;11:75. doi: 10.3389/fnbeh.2017.00075 (PMC5413561; doi:10.3389/fnbeh.2017.00075)
Supplement: Supplementary file 1 [file Data_Sheet_1.docx]

Supplementary Material

Selegiline Ameliorates Depression-Like Behavior in Mice Lacking the *CD157/BST1* Gene, a Risk Factor for Parkinson’s Disease

Satoka Kasai^1, 2*^, Toru Yoshihara^2, 3^, Olga Lopatina^2^, Katsuhiko Ishihara^4^ and Haruhiro Higashida^2*^

***Correspondence:**Haruhiro Higashida, Research Center for Child Mental Development, Kanazawa University, 13-1 Takara-machi, Kanazawa 920-8640, Japan
[haruhiro@med.kanazawa-u.ac.jp](mailto:haruhiro@med.kanazawa-u.ac.jp)
Satoka Kasai, Research Institute, FP Pharmaceutical Corporation, 1-3-40 Nishiotsuka, Matsubara, Osaka 580-0011, Japan
[soyaku@fp-pharm.co.jp](mailto:soyaku@fp-pharm.co.jp)


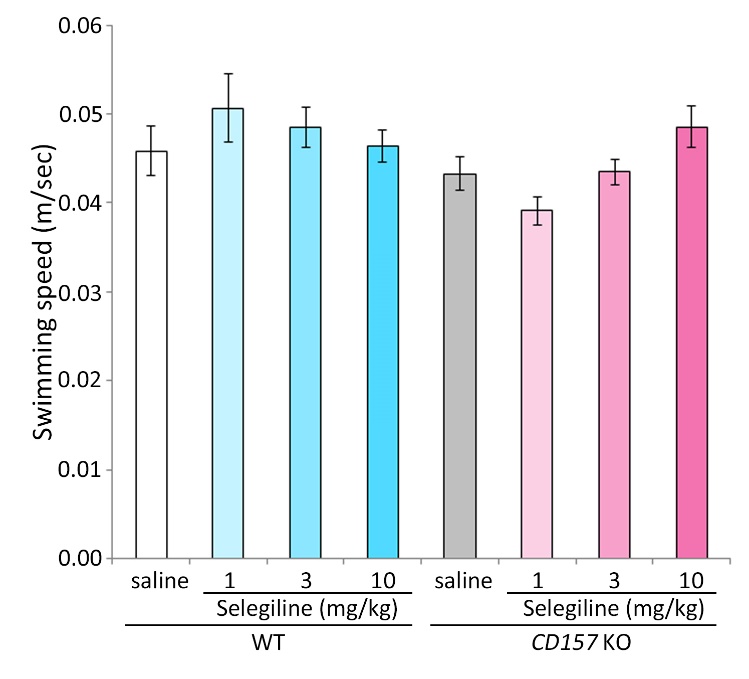


**Supplementary Figure**

**Effects of selegiline on swimming speed of WT and *CD157* KO mice in the FST**

There were no differences in swimming speed between genotypes or treatment groups. The data are expressed as the mean ± SEM (n = 21−22 for saline-treated WT and *CD157* KO mice, n = 15−17 for selegiline-treated WT and *CD157* KO mice). A two-way ANOVA showed no significant interaction between the effects of treatment and genotypes on the swimming speed [*F*_(3,128)_ = 2.671, *P* = 0.050].
